# Genomics of *Ochrobactrum pseudogrignonense* (newly named *Brucella pseudogrignonensis*) reveals a new *bla*_OXA_ subgroup

**DOI:** 10.1099/mgen.0.000626

**Published:** 2021-08-27

**Authors:** Shu-Yuan Li, Yin-En Huang, Jhih-Yang Chen, Chung-Hsu Lai, Yan-Chiao Mao, Yao-Ting Huang, Po-Yu Liu

**Affiliations:** ^1^​ Division of Infectious Diseases, Department of Internal Medicine, Taichung Veterans General Hospital, Taichung, Taiwan, ROC; ^2^​ Department of Computer Science and Information Engineering, National Chung Cheng University, Chia-Yi, Taiwan, ROC; ^3^​ Division of Infectious Diseases, Department of Internal Medicine, E-Da Hospital, Kaohsiung, Taiwan, ROC; ^4^​ School of Medicine, College of Medicine, I-Shou University, Kaohsiung, Taiwan, ROC; ^5^​ Department of Emergency Medicine, Division of Clinical Toxicology, Taichung Veterans General Hospital, Taichung, Taiwan, ROC; ^6^​ School of Medicine, National Defense Medical Center, Taipei, Taiwan, ROC; ^7^​ Rong Hsing Research Center for Translational Medicine, National Chung Hsing University, Taichung, Taiwan, ROC; ^8^​ Ph.D. Program in Translational Medicine, National Chung Hsing University, Taichung, Taiwan, ROC

**Keywords:** β-lactamases, *Brucella pseudogrignonensis*, *Ochrobactrum pseudogrignonense*, OXA

## Abstract

*
Ochrobactrum pseudogrignonense* (newly named *Brucella pseudogrignonensis *) is an emerging pathogen in immunodeficient and immunocompetent patients. Most documented cases associated with *Ochrobactrum* are frequently catheter-related and exhibit wide-spectrum β-lactam resistance. Misidentification of this pathogen using commercial bacterial identification kits is common. We identified a case of *O. pseudogrignonense* infection associated with cholelithiasis. The *O. pseudogrignonense* genome was sequenced and reconstructed using a Nanopore and Illumina hybrid strategy. A novel *bla*_OXA-919_ divergent from existing OXA members was identified and subsequent analysis revealed its existence in all available *O. pseudogrignonense* genomes, which forms a new phylogenetic subgroup distinct from other OXA clusters. Further analysis demonstrated the presence of the novel *bla*_OXA-919_ in the chromosome of several other *Ochrobactrum* species. Our study indicated that *Ochrobactrum* chromosomes may be a reservoir of *bla*_OXA-919_ β-lactamases.

## Data Summary

All the sequencing data have been deposited in GenBank under BioProject ID no. PRJNA505957 accession PKQI00000000. (https://www.ncbi.nlm.nih.gov/nuccore/PKQI00000000.1/). All the supporting data have been provided through supplementary data files.

**Impact Statement**

*
Ochrobactrum pseudogrignonense* (newly named *Brucella pseudogrignonensis *) is an emerging pathogen exhibiting wide-spectrum β-lactam resistance, but the underlying resistance determinants are as yet unclear. By sequencing, reconstructing and comparing the *O. pseudogrignonense* genomes, we found a novel subgroup of OXA β-lactamases, OXA-919, which is found not only in *O. pseudogrignonense* genomes but also in other *Ochrobactrum* species. Our results revealed a new OXA cluster.

## Introduction

*Ochrobactrum* has been recognized as an emerging pathogen in immunodeficient and immunocompetent patients. Recently, the genus *Ochrobactrum* was renamed within *Brucella *. However, the clinical characteristics are different between the two groups. To date, *Ochrobactrum anthropi* (newly named *B. anthropi *), *O. intermedium* (*B. intermedium*), *O. tritici* (* B. tritici *), *O. haematophilum* (*B. haematophilum*) and *O. pseudogrignonense* (* B. pseudogrignonensis *) have been reported to cause human infections [1]. Most *Ochrobactrum* infections are catheter-related, such as central venous vein catheters, drainage tubes and intraperitoneal catheters, because of the ability of the pathogen to adhere to silicone [1]. *Ochrobactrum* species show wide-spectrum β-lactam resistance. Previous studies reported that β-lactam resistance is associated with production of an inducible chromosomally encoded Amp-C β-lactamase [2]. Misidentification of *Ochrobactrum* species using commercial bacterial identification kits is common and leads to diagnostic difficulties in clinical settings [1].

*O. pseudogrignonense* is a gram-negative, non-motile, non-spore-forming and oxidase-positive rod-shaped bacterium [3]. It is a naturally occurring environmental organism found in water and soil [4]. It has been shown to be a pathogen of a fungus [5]. Whole genome sequencing of *O. pseudogrignonense* was reported in 2016 from Malaysian tropical soil. Clinical cases were reported in Sweden in 1992 and in Norway in 2000, respectively, from blood of a 28-year-old patient and the ear of a newborn [3]. Recently, a human case report in Korea described bacteraemia in a 44-year-old man with extracorporeal membrane oxygenation [6].

In this study, we report the genomic analysis of a clinical *O. pseudogrignonense* isolate in Taiwan and conduct comparative genomics with publicly available genomes of *O. pseudogrignonense* (K8, MYb58, CCUG 30717, CCUG43892, MYb37, MYb70 and CIP 109451).

## Methods

### Sample description

An 86-year-old male patient presented with fever and abdominal pain. He had a background of hypertension, history of liver abscess, benign prostatic hyperplasia and pulmonary tuberculosis (for which he received complete treatment at age of 20 years). He received percutaneous transhepatic gallbladder drainage under the impression of gallstone-related acute cholecystitis. After drainage, fever improved. Liver sonography showed bilateral intrahepatic duct dilatation. T-tube cholangiography revealed an obstructive cystic duct. He was referred to a general surgeon and was admitted to the ward for laparoscopic cholecystectomy.

On the first day of hospitalization, he was afebrile, with blood pressure 133/71 mmHg and heart rate 72 b.p.m. Laboratory findings indicated a white blood cell count of 6700 μl^–1^ with a differential of 60 % neutrophils, a haemoglobin level of 11.5 g dl^−1^, a platelet count of 183×10^3^ μl^–1^, total bilirubin of 0.3 mg dl^−1^, direct bilirubin of 0.1 mg dl^−1^, AST (aspartate aminotransferase) of 18 U l^–1^ and ALT (alanine aminotransferase) of 8 U l^–1^. He received laparoscopic cholecystectomy on admission day 2. Intravenous antibiotics with cefazolin (1000 mg every 8 h) was started on the day of operation. Operative findings showed gallbladder about 6×13 cm in size with multiple pigmented stones inside and a cystic duct diameter of about 0.84 cm. Bile leakage was observed during the operation, so he received wound care with subsequent wet dressing. The wound was closed on post-operative day 2. Brown colour drainage was observed on post-operative day 3. Because bile leakage was suspected, he received endoscopic retrograde biliary drainage on post-operative day 5. Surgical pathology showed cholelithiasis and chronic active cholecystitis. On post-operative day 5, bile culture yielded morphologically homogeneous colonies which were preliminarily identified as *O. anthropi* by MALDI-TOF. He received intravenous tigecycline (100 mg loading dose, maintenance dose 50 mg every 12 h) for 7 days and was discharged on day 14. He received biliary drainage removal 2 months after discharge without specific complications.

Whole genome sequencing of the isolate and computation of whole-genome average nucleotide identity (ANI) against other species in the same genus was performed. The results indicated 98.99 % ANI with *O. pseudogrignonense* CCUG 30717. Antimicrobial susceptibility testing showed that the isolate was sensitive to imipenem, colistin, tigecycline, amikacin and trimethoprim-sulfamethoxazole, but resistant to gentamicin, ampicillin-sulbactam, ceftazidime, cefepime, ceftriaxone, ciprofloxacin and piperacillin-tazobactam.

### Genome sequencing. assembly and annotation

The *O. pseudogrignonense* SHIN genome was deeply sequenced using Nanopore long-read sequencing at 123× and Illumina short-read sequencing at 56×. Adaptor sequences left in long reads were trimmed using Porechop. The remaining reads were hybrid-assembled via Unicycler (v0.4.7) (N50=1 880 620 bp), and classified into two chromosomes and three plasmids. Protein-coding genes, and coding and non-coding RNAs in the chromosomes and plasmids were annotated via the NCBI PGAP pipeline. Antibiotic-resistant genes (ARGs) were predicted by aligning protein-coding genes against the Comprehensive Antibiotic Resistance Database (CARD) using Diamond. Only ARGs with alignment coverage greater than 90 % were retained. Efflux pumps were excluded from ARG analysis.

### Motif and phylogenetic analysis of OXA family members

Multiple sequence alignment of OXA-919 and other OXA members was carried out by mega x to identity the conserved motifs. The alignment was used for generating a phylogeny tree by mega x, which was visualized by ITOL in order to construct a circular phylogeny tree.

### Data deposition

This Whole Genome Shotgun project has been deposited at DDBJ/ENA/GenBank under accession PKQI00000000. The version described in this paper is version PKQI00000000.1.

## Results

### Hybrid genome sequencing revealed beta lactamases of multiples classes

**Fig. 1.**
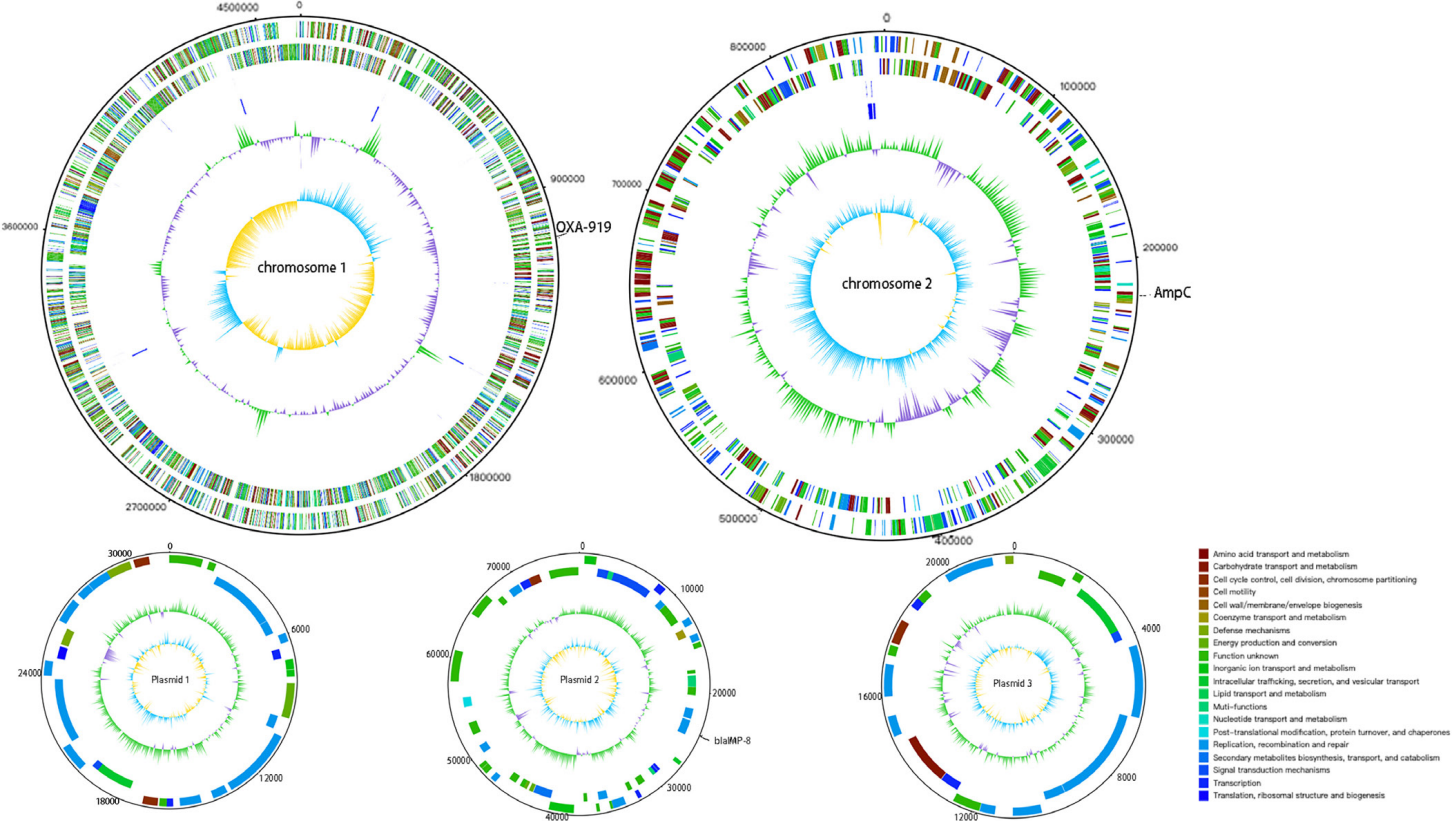
Circular maps of two chromosomes and three plasmids of *O. pseudogrignonense* (newly named *B. pseudogrignonense*) SHIN. The novel *bla*_OXA-919_ is on chromosome 1, *bla*_Imp-8_ is on plasmid 2 and ampC is on chromosome 2.

The *O. pseudogrignonense* SHIN genome was sequenced by Nanopore (123×) and Illumina (56×) (Table S1, available in the online version of this article). Two chromosomes and three plasmids were obtained (Fig. 1, Table S2). The genome encompasses 5199 protein-coding genes, 15 5S/16S/23S rRNAs, 62 tRNAs and four non-coding RNAs. *O. pseudogrignonense* SHIN was resistant to a wide spectrum of β-lactams, including penicillin, cephalosporins and carbapenems (Table S3). Analysis of the SHIN resistome indicates the presence of 148 ARGs, including three β-lactamases in distinct classes (Table S4). In particular, one novel class D β-lactamase (called *bla*_OXA-919_) is found on chromosome 1. There are no associated mobile genetic elements or integrative and conjugative elements found around *bla*_OXA-919_. A class C β-lactamase (ampC) is present on chromosome 2. A class B metallo-beta-lactamase, *bla*_Imp-8_, is carried by plasmid 2 (Fig. S1). These chromosomal-encoding β-lactamases along with plasmid-encoding carbapenemase are probably the major resistance factors to a wide spectrum of β-lactams.

### The novel class D *bla*_OXA-919_ beta lactamase is harboured by all *O. pseudogrignonense*

**Fig. 2.**
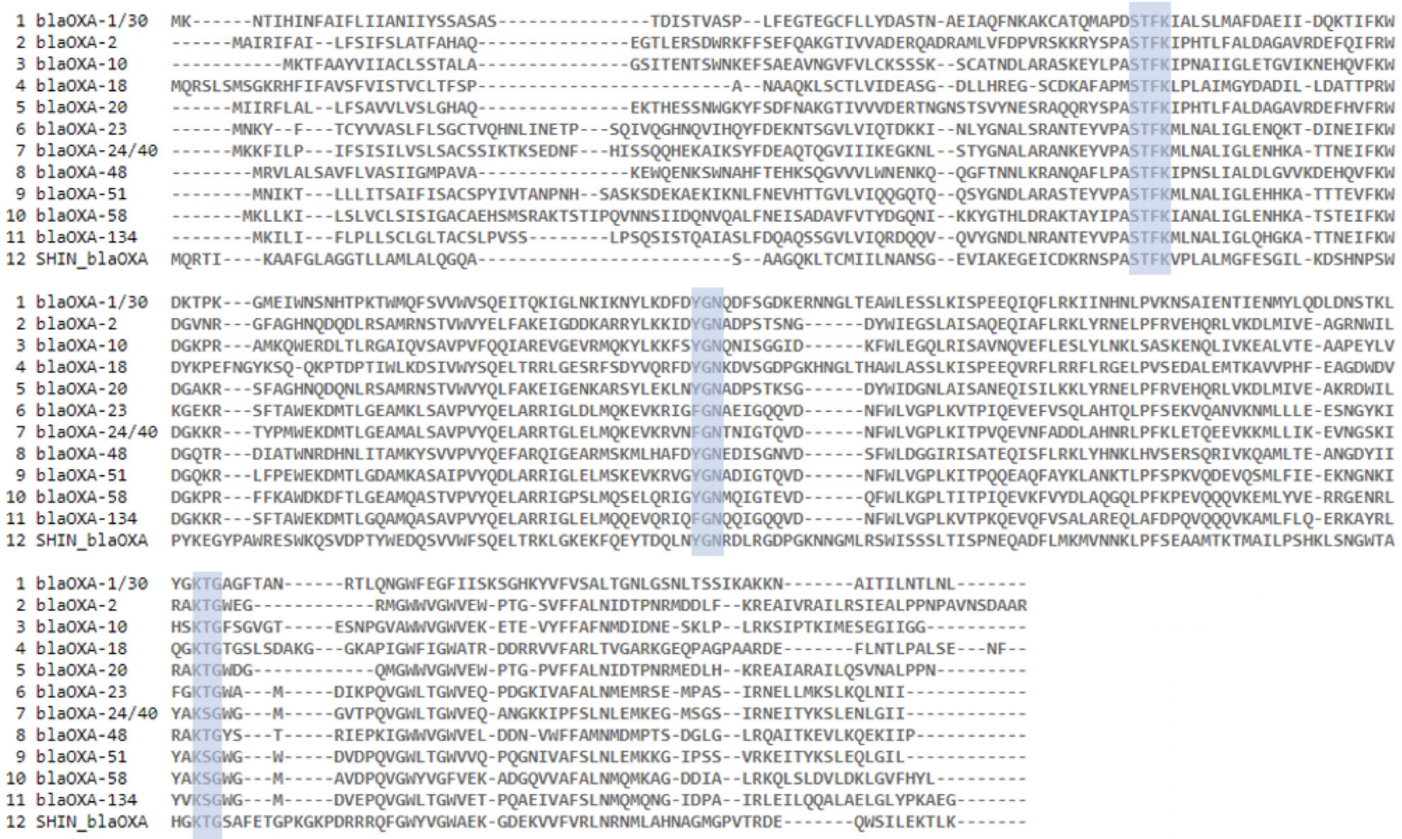
Comparison of conserved motifs within OXA members (blue marks). The S-T-F-K, Y-G-N and K-T-G motifs are highly conserved in class D β-lactamases, although the F-G-N and K-S-G motifs may replace Y-G-N and K-T-G in several OXAs. The new *bla*_OXA-919_ also exhibits the same high conservation.

The class C β-lactamase, ampC, is well known in *Ochrobactrum* genomes and also encoded in chromosome 2 of the SHIN genome. To the best of our knowledge, this is the first report of the presence of class D β-lactamases (OXA) in *Ochrobactrum* (designated as NG_070746.1/WP_007879679.1 by NCBI). By comparing *bla*_OXA-919_ with known OXA members, we found that it is quite dissimilar from others (e.g. AAI=40.16% to OXA-45 and AAI=27.52 % to OXA-54). Nevertheless, *bla*_OXA-919_ contains classic OXA signatures of three highly conserved motifs, S-T-F-K, Y-G-N and K-T-G, although the sequence outside the motifs diverges (Fig. 2), implying it is possibly a novel member in the current OXA family. We then used a blast search for the presence of *bla*_OXA-919_ in other public *O. pseudogrignonense* genomes in NCBI (Table S5) and found all of them carry *bla*_OXA-919_ (Table S5). By searching the NCBI protein database, *bla*_OXA-919_ (WP_007879679.1) is 100 % identical to one annotated protein (AKVI01000111.1/EMG52215.1) in the *Ochrobactrum* sp. CDB genome. This indicated that, in addition to ampC, *bla*_OXA-919_ is another common β-lactamase encoded under the genus *Ochrobactrum *. Consequently, *Ochrobactrum* is probably the reservoir of class C and class D β-lactamases. Unfortunately, the resistance profiles of these public *Ochrobactrum* genomes are not available. Therefore, we investigated the resistance phenotypes of 13 additional *Ochrobactrum* samples collected in our hospital (Table S6), including nine *O. anthropi* and four *O. intermedium *. All of these additional samples exhibited strong resistance to penicillin, and first- and third-generation cephalosporins.

### *bla*_OXA-919_ forms a new cluster within the OXA phylogeny

**Fig. 3.**
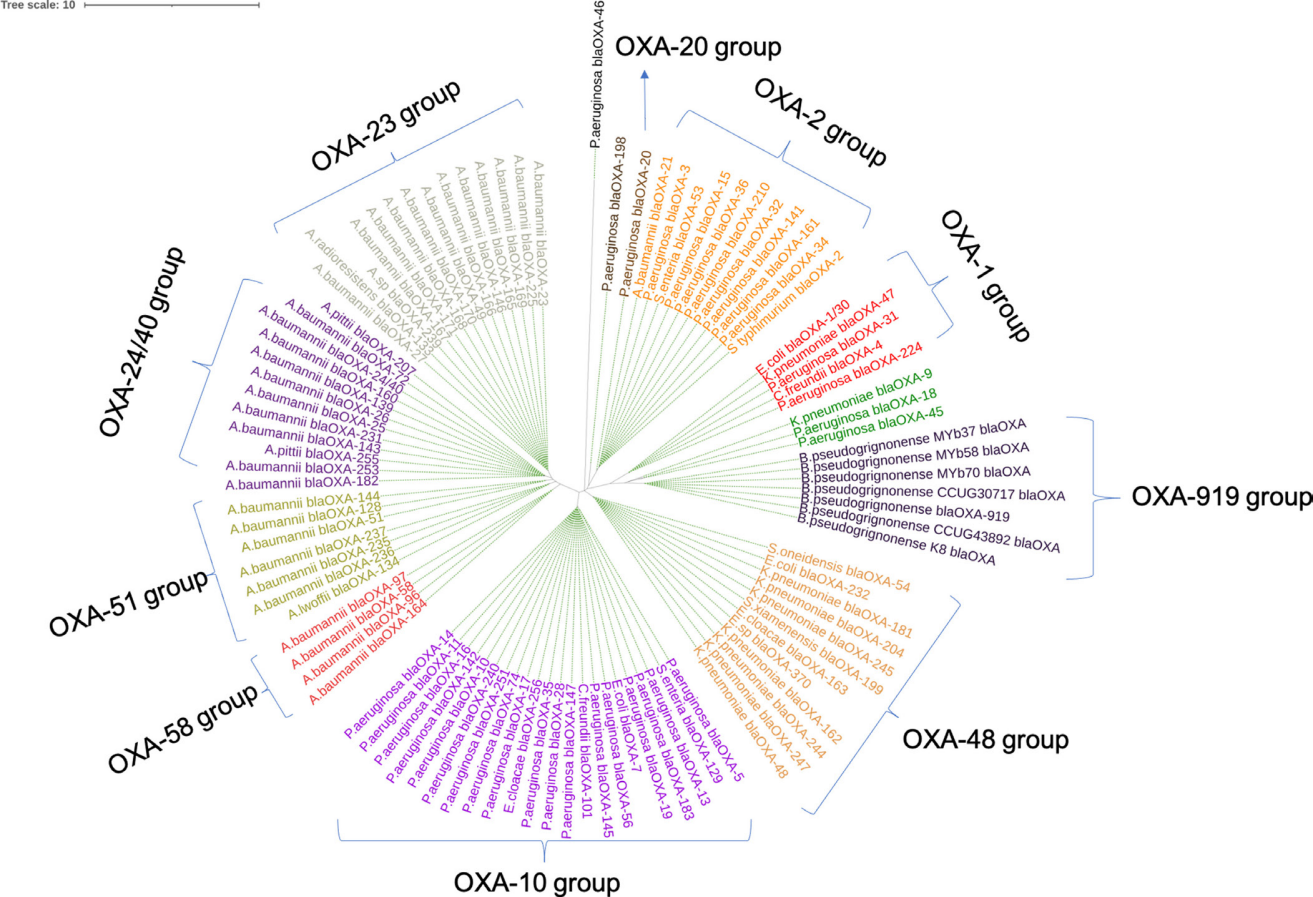
Circular phylogeny of ten OXA groups. The novel *bla*_OXA-919_ allele is clustered with other highly similar members (> 98.5 % AAI) within *B. pseudogrignonense* (formerly *O. pseudogrignonense *). These *bla*_OXA-919_ alleles form a new subgroup between he OXA-1 and OXA-48 groups.

Phylogenetic analysis of *bla*_OXA-919_ sequences from the seven *O. pseudogrignonense* genomes with existing nine-group OXA members revealed a new group (called *bla*_OXA-919_ group) (Fig. 3). The *bla*_OXA-919_ alleles from *O. pseudogrignonense* are highly similar to each other (> 98.5 % AAI) (Fig. S2). The resulting *bla*_OXA-919_ group falls between the OXA-1 and OXA-48 groups, implying an OXA cluster is missing from the existing phylogeny. Comparison of the GC content of an OXA gene with that of the whole genome provides clues to the origin of the gene because their GC contents are likely to be similar. The GC content of *bla*_OXA-919_ is 52.66 % in the SHIN genome, which is similar to that of whole genomes of *O. pseudogrignonense* (53.6 –54.32 %) (Table S7). The GC content of *bla*_OXA-919_ is also quite different from other OXA members (e.g. 37.5 % in OXA-23), suggesting that *bla*_OXA-919_ may have existed in *O. pseudogrignonense* for a long period of time. Analysis of OXA-919 of 17 *Ochrobactrum* species indicated that OXA-919 is also found in several species (e.g. *O. quorumnocens*, *O. pituitosum *) but not in others (e.g. *O. anthropi *, *O. cytisi *) (Table S8).

## Discussion

*Ochrobactrum* species are emerging pathogens in immunodeficient and immunocompetent patients [7]. We report the first case of *O. pseudogrignonense* infection and whole genome sequencing in Taiwan.

*Ochrobactrum* infections are associated with catheters or direct contamination of wounds [8–10], intravenous fluid [4], grafts [11] and medical devices [12, 13] by the pathogen. Case reports of infections related to *Ochrobactrum* species have included endocarditis [14–16], meningitis [11], brain abscess [8], peritonitis [17, 18], endophthalmitis [12, 19], osteomyelitis [10], prostatitis [13], septic arthritis [9], urosepsis [20], soft tissue infection [21], pancreatic abscess [22] and pneumonia [23].

A literature review revealed 15 studies associated with biliary tract or gastrointestinal tract infection caused by *Ochrobactrum* species. The isolated pathogen included *Ochrobactrum tritici *, *O. anthropi* and *O. intermedium* (Table 1). Most cases had underlying chronic illness or malignancy and the patient received a procedure during admission (biliary drainage, operation, post-operative drainage tube and endoscopic retrograde cholangiopancreatography) before infection episodes. A pathogen from the environment and adhering to the catheter may be the possible source of infection, similar to most previous case reports. Three cases [18] reported biliary sepsis and spontaneous bacterial peritonitis (SBP) without a drainage tube, demonstrating the possibility of *Ochrobactrum* species originating from the human gastrointestinal tract or biliary tract. Isolated culture from stool before bacteraemia [24] and antral biopsy [25] supported a possible source from the gastrointestinal tract. *Ochrobactrum* has been proposed as a component of the normal human intestinal flora and pathogen of a fungus tumour in the literature. Bacterial translocation from the gastrointestinal tract or acquisition from the mouth may be the possible route of pathogenesis of *Ochrobactrum* infections. Most cases recovered from *Ochrobactrum* infection, even under inappropriate empirical antibiotics, support the low pathogenicity of *Ochrobactrum* species.

**Table 1.** Reported cases of intra-abdominal infections by *Ochrobactrum* species

Case no. (Ref.)	Age (years)/sex	Underlying conditions	Clinical presentation	Indwelling catheter/procedure	Specimens	Identification methods	Reported pathogens	Antimicrobial therapy	Outcome
1 (our case)	86/M	HTN, liver abscess, TB	Acute cholecystitis	Biliary drainage tube	Bile	Whole genome sequencing	* O. pseudogrignonense* (newly named *B. pseudogrignonense*)	TGC	Recovered
2 [36]	70/M	CCC	Bacteraemia, cholecystitis	Biliary drainage tube	Bile, blood	16S rRNA partial sequencing	* O. tritici *	CPZ/SUL	Recovered
3 [18]	62/M	Pancreatic cancer	Biliary sepsis	No	Blood	Vitek II∗	* O. anthropi *	ERP	Recovered
4 [18]	60/M	CCC	Biliary sepsis	Biliary drainage tube	Bile, blood	Vitek II∗	* O. anthropi *	IMP	Recovered
5 [18]	70/M	CCC	Bacteraemia, cholecystitis	Biliary drainage tube	Bile	Vitek II∗	* O. anthropi *	PIP/TAZO†	Recovered
6 [18]	57/M	CCC	Biliary sepsis	Biliary drainage tube	Bile, blood	Vitek II∗	* O. anthropi *	IMP	Recovered
7 [18]	57/M	HCC	Biliary sepsis	Biliary drainage tube	Blood	Vitek II∗	* O. anthropi *	IMP	Recovered
8 [18]	69/M	CCC	Biliary sepsis	Biliary drainage tube	Bile, blood	Vitek II∗	* O. anthropi *	CIP	Recovered
9 [18]	67/M	CCC	Biliary sepsis	Biliary drainage tube	Blood	Vitek II∗	* O. anthropi *	CTX+MTZ†	Recovered
10 [18]	58/F	HCC	Biliary sepsis	No	Blood	Vitek II∗	* O. anthropi *	CTX†	Recovered
11 [18]	62/M	Cirrhosis	SBP	No	Blood, ascites	Vitek II∗	* O. anthropi *	IMP	Died‡
12 [25]	26/M	None	Non-ulcer dyspepsia	No	Antral biopsy	16S rRNA+RecA gene sequencing	* O. intermedium *	(−)	(−)
13 [37]	74/M	Bladder cancer	Bacteraemia	Elective exploratory laparotomy, colostomy	Blood, stool	16S rDNA sequencing+phenotype test§	* O. intermedium *	IMP +CIP	Recovered
14 [24]	45/F	PSC with Child-Pugh A cirrhosis	Cholangitis, liver abscess	Orthotopic liver transplantation+Roux-en-Y hepaticojejunostomy	Blood, stool (before and during bacteraemia), abscess culture (OP)	16S rDNA primers followed by DNA sequence analysis	* O. intermedium *	IMP+CIP re-transplant	Recovered
15 [38]	61/F	HTN, CKD, MI, old CVA, gallstone pancreatitis post-cholecystectomy	Cholangitis after ERCP	ERCP and T tube	T tube, blood culture	Unknown	* O. anthropi *	GEM+IMP → TMP/SMZ+CAZ	Died||
16 [22]	75/M	Chronic lung disease, HTN, MI, stroke	Pancreas abscess+gastric outlet obstruction	Laparotomy+external pancreatic drainage+side to side gastro-jejunostomy	Pancreas abscess (OP)	Unknown	* O. anthropi *	GEM	Died¶

∗Vitek II (bioMérieux).

†Non-susceptible antibiotic.

‡Due to gastrointestinal bleeding.

§Phenotype test: resistance to both colistin/polymyxin B.

||Due to progressive liver failure.

¶Due to aspiration.

**HTN, hypertension; TB, tuberculosis; CCC, cholangiocarcinoma; HCC, hepatocellular carcinoma; CKD, chronic kidney disease; MI, myocardial infarction; CVA, cerebrovascular accident; SBP, spontaneous bacterial peritonitis; PSC, primary sclerosis cholangitis; ERCP, endoscopic retrograde cholangiopancreatography.

††TGC, tigecycline; CPZ/SUL, cefoperazone/sulbactam; ERP, ertapenem; IMP, imipenem; PIP/TAZO, piperacillin/tazobactam; CIP, ciprofloxacin; CTX, cefotaxime; MTZ, metronidazole; GEN, gentamicin; TMP/SMZ, trimethoprim/sulfamethoxazole; CAZ, ceftazidime.

Misidentification of the pathogen using commercial bacterial identification kits also occurred in our case and the literature (Table 2). Vaidya *et al*. [26] reported that a case of pelvic abscess due to *O. intermedium* was incorrectly identified as *O. anthropi* by API 20NE and 16S RNA gene sequencing analysis. Misidentification of *Brucella* species as *O. anthropi* using MALDI-TOF MS and the VITEK 2 system was also reported previously [27, 28]. Teyssier *et al*. [7], who tested the ability of commercial identification systems such as API 20NE and VITEK 2 to identify *Ochrobactrum* species, showed that commercial kits were not always reliable for genus and species identification. Previous studies reporting the predominant role of *O. anthropi* in human disease by using non-discriminatory methods and/or before the discovery of other *Ochrobactrum* species suggested that some infections associated with *O. anthropi* in the literature should be revised. Further phenotypic features and genotyping methods (ex. *recA*-based analysis [29]) should be considered for more reliable identification of *Ochrobactrum* to the species level.

**Table 2.** Identification issues in clinical reports of *Ochrobactrum* infection

Case no. (Ref.)	Methods for initial identification	Initial identification	Methods for re-identification	Revised identification
1 (our case)	Vitek 2	* Ochrobactrum anthropi *	Whole genome sequencing	* Ochrobactrum pseudogrignonense* (newly named *Brucella pseudogrignonense*)
2 [36]	MALDI-TOF MS	* Ochrobactrum anthropi *	16S rRNA partial sequencing	* Ochrobactrum tritici *
13 [37]	Vitek	* Ochrobactrum anthropi *	16S rDNA sequencing+resistance to both colistin/polymyxin B	* Ochrobactrum intermedium *
14 [24]	API 20 NE system	* Ochrobactrum anthropi *	16S rDNA primers followed by DNA sequence analysis	* Ochrobactrum intermedium *

Most *Ochrobactrum* spcies are resistant to most β-lactams and sensitive to carbapenem, ciprofloxacin, trimethoprim/sulfamethoxazole or aminoglycoside (Table 3) [30]. Hence, empirical treatment with carbapenem, quinolone, cotrimoxazole and aminoglycoside is feasible. *Ochrobactrum* exhibited resistance to β-lactam antibacterial agents due to production of an inducible and chromosomally encoded Amp-C β-lactamase [2]. The β-lactamase characterized from *O. anthropi* was named OCH-1 (gene, *bla*_OCH-1_) [31]. Alonso *et al*. found the *bla*_OCH_ gene in non-* O. anthropi* species with gene heterogeneity from food animals [32]. The isolated species identified by 16S rDNA sequencing and MALDI-TOF MS included *O. intermedium* and *O. tritici *. However, there is limited data regarding antibiotic resistance genes in non-*antropi Ochrobactrum* species from humans.

**Table 3.** Antimicrobial susceptibility profiles in clinical reports of *Ochrobactrum* infections

Case no. (Ref.)	AMP/ SUL	PIP/ TAZO	CRO	CAZ	FEP	CIP	GEM	AMK	TMP/SMZ	CR	TGC
1 (our case)	R	R	R	R	R	R	R	S	S	S	S
2 [36]			R		R						
3 [18]		R	R	R	R	S	I	R	S	S	
4 [18]		R	I	R	I	S	S	S	S	S	
5 [18]		R	I	R	R	S	I	I	S	S	
6 [18]		R	R	R	R	S	I	I	S	S	
7 [18]		R	R	R	R	S	S	S	S	S	
8 [18]		R	R	R	R	S	S	S	S	S	
9 [18]		R	R	R	R	S	S	S	S	S	
10 [18]		R	R	R	R	S	S	S	S	S	
11 [18]		R	R	R	R	S	S	S	S	S	
12 [25]						S	R	R	S	R	S*
13 [37]						S	S		S	S	
14 [24]				R (>256)		S (0.19)			S†	S‡	
15 [38]	No data available										
22]							S	R	S		R*

*Test tetracycline.

†Blood 0.094µg ml^−1^, liver/faeces 0.125 µg ml^−1^.

‡Imipenem, blood 1.5 µg ml^−1^, liver/faeces 1.0 µg ml^−1^.

§AMP/SUL, ampicillin/sulbactam; PIP/TAZO, piperacillin/tazobactam; CRO, ceftriaxone; CAZ, ceftazidime; FEP, cefepime; CIP, ciprofloxacin; GEM, gentamicin; AMK, amikacin; TMP/SMZ, trimethoprim/sulfamethoxazole; CR, carbapenem; TGC, tigecycline.

A new *bla*_OXA_, OXA-919, was found in our isolate and confers resistance to broad-spectrum β-lactam antibiotics. The emergence of OXA enzymes in recent years has caused huge difficulty in treating gram-negative infections. Some intrinsic OXAs have been identified in *Acinetobacer* species [33]. OXA-51-like β-lactamases are intrinsic to *Acinetobacter baumannii* [34] and may cause carbapenem resistance. OXA-134a is universal in *Acinetobacter lwoffii* [35] and may have the potential to cause β-lactam resistance when transferred to other species. We analysed seven *O. pseudogrignonense* genomes and found all *O. pseudogrignonense* isolates harboured *bla*_OXA-919_ genes. Further investigation identified *bla*_OXA-919_ genes in several other *Ochrobactrum* species. *Ochrobactrum* may be a reservoir of OXA-919.

*O. pseudogrignonense* is an emerging pathogen in immunodeficient and immunocompetent patients. Clinical isolates exhibit wide-spectrum β-lactam resistance. Whole genome sequencing shows that Class B, C and D β-lactamases are present in the SHIN genome. A new subgroup of OXA, OXA-919, is identified. *O. pseudogrignonense* may be a reservoir of multiple β-lactamases, and the impact on antibiotic resistance transfer needs further study.

## Supplementary Data

Supplementary material 1

Supplementary material 2
