# Supplementary material for: Genomics of Ochrobactrum pseudogrignonense (newly named Brucella pseudogrignonensis) reveals a new bla OXA subgroup
Source: Microb Genom. 2021 Aug 27;7(8):000626. doi: 10.1099/mgen.0.000626 (PMC8549353; doi:10.1099/mgen.0.000626)
Supplement: Supplementary material 1 [file mgen-7-0626-s001.pdf]

Figure S1

K.P. blaIMP-8

O.P. SHIN blaIMP-8

```
tr|Q9AMR1|Q9AMR1_KLEPN      MKKLFVLCVCFLCSITAAGAALPDLKIEKLEEGVYVHTSFEEVNGWGVVSKHGLVVLVNT      60
SHIN_blaIMP                 MKKLFVLCVCFLCSITAAGAALPDLKIEKLEEGVYVHTSFEEVNGWGVVSKHGLVVLVNT      60
                            ************************************************************

tr|Q9AMR1|Q9AMR1_KLEPN      DAYLIDTPFTATDTEKLVNWFVERGYKIKGTISSHFHSDSTGGIEWLNSQSIPTYASELT      120
SHIN_blaIMP                 DAYLIDTPFTATDTEKLVNWFVERGYKIKGTISSHFHSDSTGGIEWLNSQSIPTYASELT      120
                            ************************************************************

tr|Q9AMR1|Q9AMR1_KLEPN      NELLKKDGKVQAKNSFSGVSYWLVKNKIEVFYPGPGHTQDNVVVWLPEKKILFGGCFVKP      180
SHIN_blaIMP                 NELLKKDGKVQAKNSFSGVSYWLVKNKIEVFYPGPGHTQDNVVVWLPEKKILFGGCFVKP      180
                            ************************************************************

tr|Q9AMR1|Q9AMR1_KLEPN      DGLGNLGDANLEAWPKSAKILMSKYGKAKLVVSSHSEIGDASLLKRTWEQAVKGLNESKK      240
SHIN_blaIMP                 DGLGNLGDANLEAWPKSAKILMSKYGKAKLVVSSHSEIGDASLLKRTWEQAVKGLNESKK      240
                            ************************************************************

tr|Q9AMR1|Q9AMR1_KLEPN      PSQPSN      246
SHIN_blaIMP                 PSQPSN      246
                            ******
```

Figure S2

	OXA-919	K8_OXA	MYb70_OXA	MYb58_OXA	MYb37_OXA	CCUG43892_OXA	CCUG30717_OXA		
OXA-919	100	-	-	-	-	-	-		95
K8_OXA	99.6	100	-	-	-	-	-		96
MYb70_OXA	98.5	98.9	100	-	-	-	-		97
MYb58_OXA	98.5	98.9	100	100	-	-	-		98
MYb37_OXA	98.5	98.9	100	100	100	-	-		99
CCUG43892_OXA	99.6	100	98.9	98.9	98.9	100	-		100
CCUG30717_OXA	100	99.6	98.5	98.5	98.5	99.6	100		
